# Real-World and Patient-Reported Outcomes of Dupilumab and Other Biological Drugs for Chronic Obstructive Pulmonary Disease—A Systematic Review

**DOI:** 10.3390/diagnostics14212390

**Published:** 2024-10-26

**Authors:** Ophir Freund, Ori Wand, Sara Kutzkel, Boaz Tiran, Irina Pumin, Inbal Friedman Regev, Liran Levy, Amir Bar-Shai

**Affiliations:** 1 The Institute of Pulmonary Medicine, Tel Aviv Sourasky Medical Center, Tel Aviv 6423906, Israel; 2 Faculty of Medicine, Tel Aviv University, Tel Aviv 6997801, Israel; 3 Division of Pulmonary Medicine, Barzilai University Medical Center, Ashkelon 7830604, Israel; 4 Faculty of Health Sciences, Ben-Gurion University of the Negev, Beer-Sheva 8410501, Israel; 5 The Sheba Lung Transplant Program, Institute of Pulmonary Medicine, Sheba Medical Center, Ramat Gan 5262000, Israel

**Keywords:** chronic obstructive pulmonary disease, biologic therapy, exacerbations, immunologic treatment, eosinophils

## Abstract

Background: Over the last few decades, the efficacy of biological therapies for COPD has been evaluated by different randomized controlled trials (RCTs). Still, the evaluation of real-world data and patient-reported outcome measures (PROMs) have not been performed in this field before. In the current work, we present a systematic literature review of the real-world data and PROMs of biological treatments for COPD. Methods: Three large databases (MEDLINE/PubMed, Scopus, and ScienceDirect) were utilized for the systematic literature review. Clinical studies (RCT, cohorts, case series/reports) assessing patients with COPD treated by any biological therapy were included. Results: The review resulted in twelve eligible studies (nine randomized controlled trials and three “real-world” case series/reports). The evaluation of PROMs in the included studies was mainly limited to the severity and burden of respiratory symptoms. Most biological therapies were associated with improved PROMs compared to the baseline, although not for the placebo. Dupilumab was the only biologic therapy with proven efficacy in RCT for both objective and subjective measures. One prior study reported patients’ self-perceived drug effects, and none evaluated patients’ perceived disease status. Only 25 patients were assessed in a real-world setting for all biologic therapies combined. Real-world data were retrospective in the form of case reports or series. Conclusions: There are limited data on patients’ experience with biological therapies for COPD. While real-world data and PROMs are missing, biases such as a placebo effect must be considered, requiring their incorporation with objective outcomes from prospective controlled trials.

## 1. Introduction

Chronic obstructive pulmonary disease (COPD) is a main cause of morbidity and mortality, which continue to increase [1]. Acute COPD exacerbation represents a major complication and is the primary driver for worse outcomes [2,3,4]. Therefore, new treatments and interventions are urgently needed with proven efficacy. Although biological therapies have revolutionized the management of severe asthma [5], this is not the case with COPD. In recent years, different high-quality trials have evaluated biological therapies for COPD, with mostly negative outcomes [6,7]. The results of the BOREAS, a randomized controlled trial (RCT) published in July 2023, were the first sign of hope for new treatment options [8]. In the BOREAS trial, dupilumab treatment was found to reduce exacerbations and symptoms and improve the quality of life in patients with COPD and type-2 inflammation. These findings were validated in a second recently published RCT, the NOTUS study [9].

While promising, various biases could lead to differences between a controlled trial setting and real-world experience [10], which is currently missing. Real-world data are defined as “data relating to patient health status and/or the delivery of health care routinely collected from a variety of sources” [11]. Such data exist in biologic therapies for indications other than COPD, with more than 5 years of experience and in thousands of patients [12,13]. Real-world data have inherited limitations based on their retrospective, observational design. Still, it could shed light on under-represented patient populations in clinical trials, assess side effects, and identify sub-populations with different treatment effects [14].

Patient-reported outcome measures (PROMs) are another important aspect when evaluating new treatments. The use of PROMs aims to collect information on health outcomes directly from patients [15]. In general, tools that assess PROMs are divided into condition-specific and generic (relevant to a variety of diseases). PROMs include health-related quality of life, symptoms, functional status, and others [16]. These aspects are key attributes of quality care and could provide additional data that cannot be obtained by other means [17]. Therefore, in recent years, PROMs have been routinely included as primary or secondary outcomes by increasing the number of studies [18]. Their incorporation has been shown to improve patient–provider communication and patient satisfaction [19]. PROMs were also shown to have prognostic value in different lung diseases [20].

Considering the above, we aimed to provide a review of the current evidence on the experience of patients with COPD treated with biological therapies in real-world settings and on PROMs from the available studies on this topic.

## 2. Materials and Methods

The aim of our study is to review the currently available data on PROMs and real-world evidence on COPD patients treated with biological therapies. Given the different treatments, study designs, PROMs selected in each trial, and our overall aim, we did not perform a meta-analysis but rather focused on each biologic treatment separately. The studies’ findings are presented separately and focus on the PROMs included and not on treatment outcomes; hence, we also did not perform publication bias or general bias analysis. The study was performed and reported in accordance to the PRISMA guidelines (Supplementary Materials).

### 2.1. Eligibility Criteria

Full-text studies in English of any clinical design (prospective or retrospective) that described the characteristics and outcomes of patients with COPD treated with a biologic therapy were included. There was no restriction on the minimum number of patients described. We excluded studies of patients with other indications than COPD for the biologic treatment (such as “asthma-COPD”).

### 2.2. Information Sources

Based on accepted guidelines, the literature search was conducted using the MEDLINE/PubMed, Scopus, and ScienceDirect databases [21]. All relevant published studies until June 2024 were reviewed for inclusion. The following search strategy was employed: ((benralizumab) OR (mepolizumab) OR (omalizumab) OR (interleukin) OR (dupilumab)) AND ((COPD) OR (chronic obstructive pulmonary disease)).

### 2.3. Selection Process

**Figure 1:**
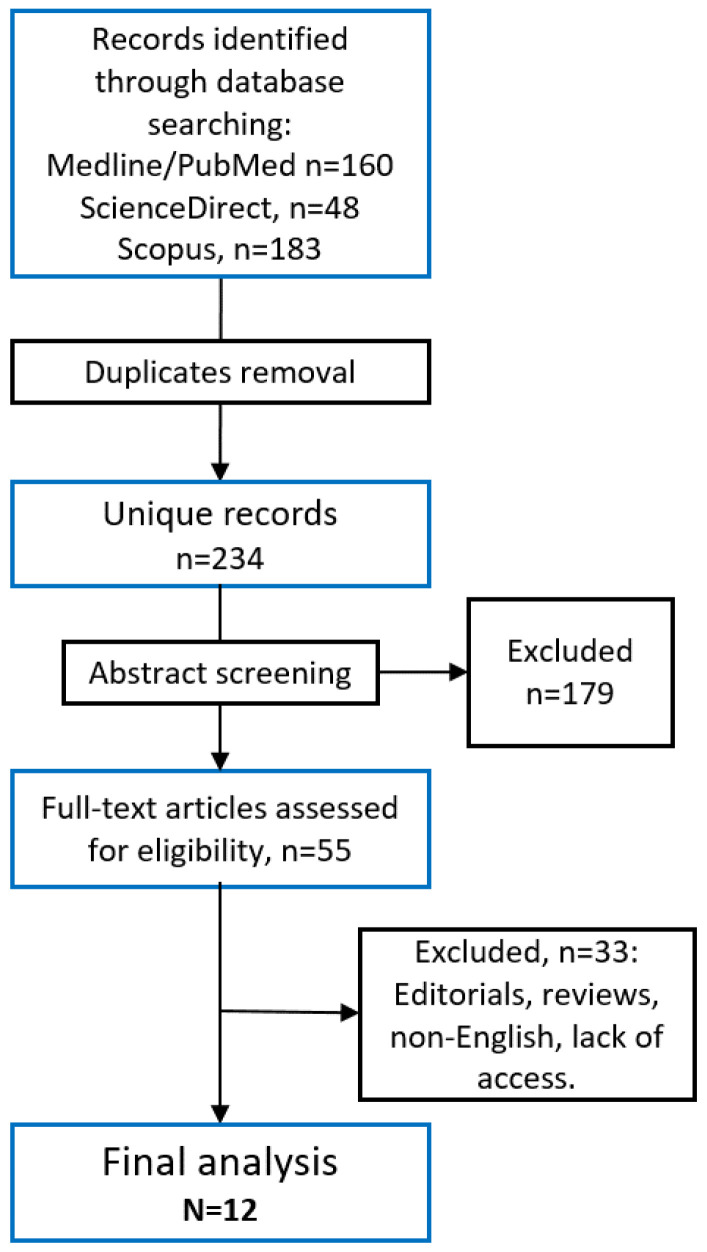
Study inclusion process.

Following the initial search, 160 articles were retrieved from MEDLINE/PubMed, 48 from ScienceDirect, and 183 from Scopus (Figure 1). The titles and abstracts of all studies were reviewed by two independent pulmonologists (S.K. and A.M.). Following this initial screening, the full text of studies that potentially comply with the inclusion/exclusion criteria were reviewed for their final decision by two independent physicians (B.T. and I.P.). In cases of disagreement, a third reviewer (A.B.S.) was included in the final decision. Using this method, 12 studies met our inclusion criteria for this review. In the presented review, we focus on patient-reported outcomes as they appear in the publications of the included trials.

### 2.4. Data Compilation and Synthesis

We derived a database for data collection, which initially included a list of potential studies, and after final inclusion/exclusion, were filled by one of the research team. The variables in the database included the type of drug, dosage, study design, population, PROMs included, other primary and secondary outcomes, results, and side effects. The data were divided per each biologic drug, and those without more than one relevant study were gathered as a single group. Following this, descriptive analyses were performed per study, with the results presented in table form and in the text.

## 3. Results

### 3.1. Tools to Assess PROMs in the Included Studies

A summary of the different tools that were used to assess PROMs in the included studies is shown in Table 1. As discussed below, the tools were fairly similar between studies and mainly assessed disease-related quality of life and symptoms. The following is a short review of the different tools for a better context of the results.

The St. George’s Respiratory Questionnaire (SGRQ) assesses health impairment in subjects with COPD or asthma [22]. It comprises 16 questions or sections that address topics such as the frequency of symptoms, current activity, and mood [23]. Lower scores indicate better health-related quality of life. The tool has been validated by multiple studies for COPD and correlates with other objective outcomes [22]. Another commonly used instrument to assess health-treated quality of life in patients with COPD is the Chronic Respiratory Disease Questionnaire [CRQ] [24]. It includes 20 items from four categories (dyspnea, fatigue, emotional function, and mastery), with higher values indicating a better response. The CRQ was validated and could be either administered by an interviewer or self-administered [25].

The burden of symptoms is a main PROM and has been assessed by multiple instruments. The COPD assessment test (CAT) is a simple method to evaluate the impact of COPD symptoms on a patient’s health [26]. In different studies, it was linked to FEV1 results, SGRQ, and response to treatment [27]. The Evaluating Respiratory Symptoms in COPD (E-RS–COPD) score represents overall respiratory symptom severity and includes 11 items that were derived from the EXACT (discussed below) [28]. The score underwent internal and external validity for COPD and, based on a literature review, corresponded well with other outcome measures [29]. Finally, the transition dyspnea index (TDI) can evaluate the change in dyspnea from the baseline, taking into account three components—functional impairment, the task, and the effort that evokes dyspnea [30]. Each component is ranked from 0 to 4, with higher scores indicating lower dyspnea. This index was first derived and validated in COPD patients, with 1-unit change as the minimum clinically important difference [31].

Exacerbations of COPD are defined clinically by the worsening of symptoms, leading to a change in treatment [1]. The EXAcerbations of Chronic Pulmonary Disease Tool (EXACT) tool was derived to standardize the assessment of patients’ conditions to better characterize exacerbations [28]. It is a 14-item daily diary designed to capture the dynamic symptoms of COPD patients. After evaluation by several studies, it was found to correlate with the severity of exacerbations, their occurrence, and their response to treatment [32].

### 3.2. Dupilumab for COPD

Dupilumab is a monoclonal antibody that binds the receptor interleukin (IL)-4 alpha to inhibit the signaling of IL-4 and IL-13 [33]. It is given subcutaneously once every two weeks at a dosage of 300 mg. The BOREAS trial included 939 patients with severe COPD and ≥300 eosinophils per microliter, randomized to receive dupilumab or a placebo [8]. PROMs in this trial are shown in Table 2 and included the following: (1) COPD impacts on quality of life, assessed by the SGRQ, (2) symptomatic burden using the E-RS score, and (3) self-reported exacerbations based on the EXACT. SGRQ scores were lower in the dupilumab group, which is an improvement that occurred from week 4 to week 52 and was higher than the minimal clinically important difference (MCID). E-RS scores also improved in the dupilumab group. Finally, the number of exacerbations based on the EXACT did not differ between groups. The exacerbation rate was the primary outcome in this trial, which was reduced in the intervention group (rate ratio 0.70, 95% CI 0.58–0.86).

The second phase 3 RCT of dupilumab was the NOTUS trial, which was published in May 2024 [9]. The study included 935 patients with overall general inclusion criteria similar to the BOREAS. Similar to the BOREAS, the main PROMs included the SGRQ, E-RS score, and the EXACT for self-reported exacerbations. While the overall SGRQ scores improved in the intervention group compared to the placebo, an improvement of more than four points, the MCID, was similar between the groups. The E-RS total score improved in both the intervention and placebo groups without differences. Exacerbations assessed by the EXACT were similar between dupilumab and placebo. Regarding the primary outcome, dupilumab led to reduced exacerbations (rate ratio 0.66, 95% CI 0.54–0.82), which was found in all subgroup analyses.

In the above trials, dupilumab was not associated with higher rates of side effects compared with placebo, and the most common were nasopharyngitis and upper respiratory tract infection. Prior research has also shown a rare side effect of symptomatic eosinophilia in patients with high baseline eosinophils [34]. Based on our literature search, no other studies assessed dupilumab therapy as an indication of COPD.

**Table 2:** Characteristics of studies included in the literature review.

Trial	Biological Therapy	Design	#	Primary Outcome	Patient-Reported Outcomes				
					Quality of Life	Symptoms	Self-Reported Exacerbations	Self-Perceived Effect	Perceived COPD Status
BOREAS trial [8]	Dupilumab	RCT	939	Reduced exacerbations	Improved SGRQ score vs. placebo	Improved E-RS score	No differences, EXACT	------	------
NOTUS trial [9]	Dupilumab	RCT	935	Reduced exacerbations	Improved SGRQ score vs. placebo	Similar improved E-RS score vs. placebo	No differences, EXACT	------	------
METREX and METREO trials [7]	Mepolizumab	RCT	1512	Exacerbations, not achieved	Similar SGRQ change vs. placebo	CAT similar to placebo	------	48–57% 52-week improvement, similar to placebo.	------
Dasgupta et al. [35]	Mepolizumab	RCT	18	Decreased sputum eosinophils	CRQ similar to placebo	CAT similar to placebo	------	------	------
Revuelta-Salgado et al. [36]	Mepolizumab	Case report	1	Decreased exacerbations	------	Improved CAT from baseline	------	------	------
Larranaga et al. [37]	Anti-IL-5	Case series	17	Decreased exacerbations	------	Improved CAT from baseline	------	------	------
Laroche at el. [38]	Anti-IL-5	Case series	7	Decreased exacerbations	------	------	------	------	------
Galathea and Terranova trials [6]	Benralizumab	RCT	2665	Exacerbations, not achieved	Similar SGRQ change vs. placebo	CAT and E-RS scores similar to placebo	No differences, EXACT-PRO tool	------	------
Brightling et al. [39]	Benralizumab	RCT	82	Exacerbations, not achieved	Similar SGRQ and CRQ changes vs. placebo	------	No differences, EXACT-PRO tool	------	------
Rabe et al. [40]	Itepekimab	RCT	343	Exacerbations, not achieved	------	------	------	------	------
Calverley et al. [41]	Anti-IL-1R1	RCT	324	Exacerbations, not achieved	Similar SGRQ vs. placebo	E-RS scores similar to placebo	------	------	------
Mahler et al. [42]	Anti-IL-8	RCT	109	Dyspnea, not achieved	Similar SGRQ vs. placebo	Transition dyspnea index	------	------	------

Abbreviations: CAT, COPD assessment test; CRQ, chronic respiratory disease questionnaire; E-RS, evaluation of a respiratory symptom; EXACT, exacerbations of Chronic Pulmonary Disease Tool; IL, interleukin; RCT, randomized controlled trials; SGRQ, St. George’s Respiratory Questionnaire. The symbol # stand for the number of participants in each trial.

### 3.3. Mepolizumab for COPD

Mepolizumab, a monoclonal humanized antibody, acts by blocking cytokine IL-5 from binding to eosinophil surface receptors [43]. It is administered subcutaneously once every 4 weeks, usually at a dosage of 100 mg. The main studies that have evaluated mepolizumab for COPD were the METREX and METREO randomized controlled trials published in 2017 [7]. In general, subjects included in these trials had ≥1 exacerbation in the prior year and were on background high-dose inhaled glucocorticoids. Eosinophil cutoffs (≥150 cells/μL at inclusion or 300 cells/μL in the previous year) were used as an inclusion criterion only in the METREO trial. PROMs included an assessment of quality of life (SGRQ), symptom burden using the CAT score [44], and patient-rated response to therapy at week 52 using a 7-point Likert scale. No changes in the SGRQ and CAT scores were found between the intervention and control groups in both studies, although all were improved compared to the baseline. Self-perceived improvement at 52 weeks was noted by 48% to 57% in the different intervention groups, although they were similar in the placebo groups. The primary outcome of reduced exacerbations did not differ between the groups in the METREX (1.49 vs. 1.52 exacerbations per year). Similarly, only a trend for reduced exacerbations was found for the 100 mg METREO group (rate ratio 0.80, 95% CI 0.65–0.98, *p* = 0.07), without a difference in the 300 mg group. In both cohorts, mepolizumab was not associated with serious adverse events, with systemic reactions and injection-site reactions in 1–3% overall.

Few additional smaller-scale studies have evaluated mepolizumab for COPD. In a single-center study, 18 patients with COPD were randomized to receive mepolizumab (*n* = 8) or a placebo (*n* = 10). Like the METREX and METREO, there were no improvements in exacerbations, CAT score, and health-related quality of life (measured by the CRQ) compared with the placebo. Unfortunately, although PROMs were numerically better over time, the analyses of these results compared to the baseline were not reported [35]. A case series by Day de Larranaga et al. described 17 patients with COPD and eosinophil counts ≥250 cells/μL treated with anti-IL-5 antibodies [37]. A reduction in the rate of moderate and severe exacerbations was noted during treatment (4.06 vs. 1.15, *p* < 0.001), with improvement in CAT scores from the baseline. A second case series by Laroche et. al [38] included seven COPD patients treated with anti-IL-5 therapies (4 mepolizumab and 3 benralizumab). In their analysis, no patient-reported outcomes were assessed, while exacerbations were reduced by 78% following treatment. Finally, Revuelta-Salgado et al. described a case report of a COPD patient with severe disease and increased blood eosinophils [36]. Following treatment with mepolizumab, the patient experienced a reduction in exacerbations and improved symptoms (as assessed by the CAT score).

### 3.4. Benralizumab for COPD

Benralizumab, an IL-5-receptor alpha-directed antibody, induces a cellular cytotoxic activity to deplete eosinophils [45]. It is administered subcutaneously every 8 weeks (every 4 weeks for the first three doses), usually at a dosage of 30 mg. Its efficacy for COPD was studied by the GALATHEA and TERRANOVA phase 3 trials [6]. These randomized controlled trials included 2665 COPD patients with frequent exacerbations and did not exclude asthma if it was not considered to contribute to a current respiratory condition. The PROMs shown in these two trials were quality of life (SGRQ), symptom burden (CAT score), symptom severity (E-RS tool), patient-reported exacerbations (EXACT), and reported nights with awakenings due to respiratory symptoms. PROMs were analyzed only for subjects with an eosinophil count ≥220 cells/μL. Compared to placebo, the SGRQ was not improved in most intervention groups, as large improvements were also seen with the placebo (−3.91 in GALATHEA and −6.863 in TERRANOVA). Other indices also improved compared to the baseline, while these changes remained similar to the placebo group. Of note, a lower number of nights with awakenings was found in the intervention groups of both studies. Regarding the primary outcome, annualized exacerbations were not reduced in TERRANOVA, while a trend for reductions was seen in the GALATHEA 100 mg group (rate ratio 0.83, *p* = 0.05). In both trials, adverse events were similar in the benralizumab and placebo groups.

The phase 2a study of benralizumab for COPD included patients with sputum eosinophilia (≥3%) and similarly did not find benralizumab to reduce COPD exacerbations [39]. PROMs in this trial included the SGRQ and CRQ scores to assess COPD impact and health-related quality of life, respectively. The EXACT-PRO tool was used to measure exacerbation-free days. Numerical, yet not statistically significant, improvements from baseline were noted for these measures compared to the placebo. With the exception of the two case series described above, we did not find any real-life studies of benralizumab in COPD.

### 3.5. Other Biological Therapies for COPD

IL-33 is a cytokine released from damaged or stressed barrier tissues that may contribute to the pathology of COPD [46]. The efficacy of itepekimab, a monoclonal anti-IL-33, was evaluated in a phase 2a trial published in 2021 [40]. The study included 343 moderate/severe COPD patients randomized to receive itepekimab (300 mg, as two subcutaneous injections every 2 weeks) or a placebo and had a negative result for the primary outcome of exacerbations, without any assessment of PROMs. Phase 3 trials of anti-IL-33 drugs (itepekimab and tozorakimab) are ongoing. IL-1 receptor 1 (IL-1R1) inhibition was also a potential strategy for COPD and was evaluated in a phase 2 trial with 324 randomized COPD patients [41]. Anti-IL-1R1 treatment did not improve PROMs compared to placebo, including the SGRQ and E-RS scores, or lead to reduced exacerbations (primary outcome). Lastly, IL-8, a chemoattractant for neutrophils, was the target of a new antibody that has been examined in symptomatic COPD patients with a component of chronic bronchitis [42]. This trial randomized 109 patients and focused on patient-reported change in dyspnea (transition dyspnea index [TDI] [30]) as the primary outcome. The SGRQ was included as a secondary outcome. No changes were found between the intervention and placebo groups in TDI over 3 months (except for the first 2-week period) or in the SGRQ score.

## 4. Discussion

In this literature review, we focus on PROMs and real-world data on biological treatments for patients with COPD. Based on our literature review, biological therapies were not associated with improved COPD-related symptoms compared to the placebo in most randomized controlled trials. Only one prior study reported patients’ self-perceived drug effects, and no one evaluated patients’ perceived disease status. Dupilumab was the only drug leading to reduced exacerbations and improved quality of life in a randomized controlled trial compared to the placebo.

The majority of biologic drugs evaluated in our review have proven efficacy for conditions associated with type-2 inflammation, such as asthma [12]. Still, the potential impact of these drugs on COPD should not be undermined, as previous research found that 20–40% of COPD patients have high type-2 inflammation (indicated by high peripheral eosinophils) [47,48]. Considering the limited treatment options and the lack of new drugs approved for COPD in recent years, additional evidence for the efficacy of biological treatments is of urgent need. This should be supported by “real-world” data to provide evidence on more specific COPD sub-groups and to address PROMs. The patient-perceived effects of a drug could be crucial given its association with treatment persistence in real life [49,50]. Still, it is rarely taken into account in clinical trials, as seen in our review. Seeing the high safety profile of biological treatments and the major symptomatic burden of COPD [51,52], one might consider an improvement in PROMs alone as a means to legitimize treatment. Still, in view of the placebo effect demonstrated in our review and the financial burden of these treatments, more objective evidence is needed.

Proven interventions for COPD are limited and have mostly remained unchanged over the last decade. This issue could explain the major improvements in PROMs seen among the placebo arm in some of the included studies in our review, as patients are keen on new therapies. Until new therapies become available, there is a need to improve adherence and compliance with already proven interventions, such as pulmonary rehabilitation and smoking cessation, that are known to improve disease outcomes; yet these are not taken by many [53]

Several other limitations should be considered in the studies presented above. First, prior 1-year exacerbations were an inclusion criterion for almost all studies. Therefore, a regression towards the mean in this patient population could have led to a decrease in exacerbations, although it mainly concerns non-controlled studies (such as in real-world studies). Second, the Hawthorne effect is another issue to be considered in prospective real-world studies. This bias is the consequence of a change in people’s behavior during trials, such as taking their inhalers more regularly, leading to an alternate reason for the improved results. Finally, we cannot draw a conclusion on the overall effect of biologics on PROMs in patients with COPD due to the lack of a meta-analysis, although considering the wide variability in study drugs and characteristics, this was not feasible.

A high peripheral eosinophil count was an eligibility criterion in some of the studies, as performed in the BOREAS trial, possibly leading to a better patient selection. An analysis of the GALATHEA and TERRANOVA studies showed that a subpopulation with elevated blood eosinophil counts and a high baseline exacerbation rate was the most likely to respond to benralizumab therapy [54]. Another post hoc analysis of these trials showed similar results for 30- and 90-day outcomes [55], further supporting our assumptions. There is still a need for additional predictors of treatment response to improve patient selection and treatment outcomes.

## 5. Conclusions

PROMs analyzed in prior research in this field are limited and are mostly correlated with primary outcomes. PROMs overall improved, although often in both placebo and intervention arms, which could be explained by the inclusion of patients who are in need of new effective and safe treatments. Real-world data were lacking, without any evidence of dupilumab. Biases such as the placebo effect, which were demonstrated by most prior RCTs in this field, highlight the main role of randomized controlled trials with objective primary outcomes. Still, adding PROMs and conducting large-scale real-world studies should be encouraged to evaluate the full impact of new treatments and improve patient care.

## Figures and Tables

**Table 1:** Patient-reported outcome measures (PROMs) used in studies of biologic therapy for COPD.

PROMs	Tool	Assessment	Clinical Utility
Health-related QOL	SGRQ	Sixteen items/questions about symptoms, limitations, and impact.	Correlates with objective measures of disease, other generic PROMs, and prognosis.
Health-related QOL	CRQ	Twenty items in 4 categories measure the physical and emotional aspects of chronic respiratory disease.	Valid for self-administration. Correlates with clinical changes in time, risk for exacerbation relapses, and other measures.
Symptoms	CAT	Eight-question, patient-administered questionnaire on respiratory symptoms, confidence, sleep, and energy.	Intended for COPD, yet validated for a wide range of respiratory diseases. It significantly correlates with other PROMs and is more feasible.
Symptoms	E-RS	Eleven items that represent overall respiratory symptom severity.	Consistent relationships with other PROMs, FEV1, and effect of treatment. Correspondence with primary outcome in large randomized controlled trials.
Symptoms	Transition dyspnea index	Five-point scale on the change in dyspnea of 3 dyspnea components—functional impairment, and the task and effort to evoke dyspnea.	A simple tool that correlates with other PROMs. A 1-unit change has been validated as a minimally clinically important difference.
Self-reported exacerbation	EXACT	A daily diary-based questionnaire with 14 items assessing symptoms and their effect.	Provide a dynamic assessment. Correlate with exacerbation severity, occurrence, and response to treatment.

Abbreviations: CAT, COPD assessment test; COPD, chronic obstructive pulmonary disease; CRQ, chronic respiratory disease questionnaire; E-RS, evaluation of a respiratory symptom; EXACT, exacerbations of Chronic Pulmonary Disease Tool; FEV1, forced expiratory volume in the first second; PROMs, Patient-reported outcome measures; QOL, quality of life; SGRQ, St. George’s Respiratory Questionnaire.

## Data Availability

All data generated or analyzed during this study are included in this article.

## Supplementary Materials

The following supporting information can be downloaded at: https://www.mdpi.com/article/10.3390/diagnostics14212390/s1, The PRISMA checklist for review articles.
